# The Indoor Localization and Tracking Estimation Method of Mobile Targets in Three-Dimensional Wireless Sensor Networks

**DOI:** 10.3390/s151129661

**Published:** 2015-11-24

**Authors:** Zixi Jia, Chengdong Wu, Zhao Li, Yunzhou Zhang, Bo Guan

**Affiliations:** 1 College of Information Science and Engineering, Northeastern University, NO. 3-11 Wenhua Road Heping District, Shenyang 110819, China; E-Mails: wuchengdong@ise.neu.edu.cn (C.W.); zhangyunzhou@ise.neu.edu.cn (Y.Z.); 2 Anshan Industrial Technology Research Institute, Harbin Institute of Technology, 192 Central Qianshan Road, Anshan High-Tech Zone, Anshan 114000, China; E-Mail: lz2906190@163.com; 3 Department of Electrical Engineering and Computer Science, Northwestern University, 2145 Sheridan Road, Evanston, IL 60208, USA; E-Mail: BoGuan2015@u.northwestern.edu

**Keywords:** WSNs, three-dimensional deployment, calibration, localization

## Abstract

Indoor localization is a significant research area in wireless sensor networks (WSNs). Generally, the nodes of WSNs are deployed in the same plane, *i.e.*, the floor, as the target to be positioned, which causes the sensing signal to be influenced or even blocked by unpredictable obstacles, like furniture. However, a 3D system, like Cricket, can reduce the negative impact of obstacles to the maximum extent and guarantee the sensing signal transmission by using the line of sight (LOS). However, most of the traditional localization methods are not available for the new deployment mode. In this paper, we propose the self-localization of beacons method based on the Cayley–Menger determinant, which can determine the positions of beacons stuck in the ceiling; and differential sensitivity analysis (DSA) is also applied to eliminate measurement errors in measurement data fusion. Then, the calibration of beacons scheme is proposed to further refine the locations of beacons by the mobile robot. According to the robot’s motion model based on dead reckoning, which is the process of determining one’s current position, we employ the $H_{\infty }$ filter and the strong tracking filter (STF) to calibrate the rough locations, respectively. Lastly, the optimal node selection scheme based on geometric dilution precision (GDOP) is presented here, which is able to pick the group of beacons with the minimum GDOP from all of the beacons. Then, we propose the GDOP-based weighting estimation method (GWEM) to associate redundant information with the position of the target. To verify the proposed methods in the paper, we design and conduct a simulation and an experiment in an indoor setting. Compared to EKF and the $H_{\infty }$ filter, the adopted STF method can more effectively calibrate the locations of beacons; GWEM can provide centimeter-level precision in 3D environments by using the combination of beacons that minimizes GDOP.

## 1. Introduction

As people’s requirements for life comfort and production security advance, the demand and extent of applications for indoor localization service (ILS) increases drastically. ILS can be applied to several main areas, such as medical monitoring, underground personnel positioning, navigation in industrial production workshop and even virtual reality in the film industry [1]. All of the application settings have the following common trait: the GPS receiver is deployed in the near-surface or indoor environment, even basements, which causes the GPS signal to attenuate and the GPS-based positioning system to fail. Thus, there is great demand for the application of positioning in the aforementioned settings.

Due to many features, such as the incomplete dependence of infrastructures, low energy consumption, relatively low cost, rapid deployment, high scalability, dense node distribution, the ability to maintain normal performance in harsh and special environments, *etc*., the research and application of ILS stands out among the traditional location acquiring manners [2]. The location information is also of vital importance to the application of WSNs to monitoring. Node localization is a prerequisite and oftentimes a problem for most WSNs and also is the premise and basis of the application of WSNs to target tracking, recognition, monitoring, and so on. In short, it is playing a huge role in the practicability of WSNs.

So far, among a mass of localization research for WSNs, most studies are on 2D positioning systems, whereas studies on 3D positioning are less frequent, but increasingly popular [3,4]. In practical applications, the sensor nodes, including beacons, are usually deployed in 3D space, not in the same horizontal plane, because the indoor items and mobile people could absorb, reflect or even block the signal transmission between beacons and nodes in most indoor layouts. Most traditional 2D localization strategies are not valid for 3D deployment. Therefore, we propose to investigate localization technology to determine the locations of nodes that are three-dimensionally deployed in indoor settings. Compared to 2D deployment, 3D deployment has more advantages in practical significance and application value [5,6,7]. In the paper, we introduce an overview of a localization system that consists of sensor nodes three-dimensionally deployed in indoor settings. Since only some beacons’ locations are known when they are stuck in the ceiling, the self-localization scheme for beacons is studied to determine the locations of all beacons by measuring the distances between beacons and the static node on the floor. To ensure the localization accuracy of beacons, a calibration algorithm is proposed to refine the location coordinates by means of a mobile robot, the details of which are shown in Section 5. Lastly, we present a geometric dilution precision (GDOP)-based optimal node selection scheme to pick the group of nodes with the minimum GDOP to position the target. To improve the localization accuracy, the GDOP-based weighting estimation method (GWEM) is proposed to fuse more information from other nodes. The above research is a completely theoretical solution for localization in 3D indoor settings, and its feasibility and validity are evaluated by simulation and experiment, shown in Section 7.

## 2. Related Work

WSN-based positioning systems in three-dimensional space have been investigated for a while. Some achievements of this prior research are typical and practical for experiments. The positioning system, called SpotON, using an RF electronic label was designed and developed by Jeffrey Hightower *et al.* [8]. The whole of the localization zones is covered by multiple base stations, and the distances between unknown moving nodes and base stations are estimated by the RSSI signal attenuation model. To calculate the location coordinates, trilateration is adopted, and the hill-climbing algorithm is used to improve the estimation accuracy.

The Bat positioning system exploited by the AT&T Institution is composed of a recognizer, a receiver and a surveillance center [9]. As the recognizer receives the signal given by the surveillance center, it will respond with an ultrasonic pulse. The receiver receives an RF signal from the surveillance center and an ultrasonic pulse from the recognizer separately and obtains the time difference of arrival (TDOA) to calculate the distances between any two of the recognizers. Then, the data are reported to the surveillance center through networks. The TDOA measurement based on the ultrasonic signal can realize the spatial localization and calculate the coordinates of the unknown nodes by using trilateration or a multilateral algorithm to improve the accuracy in positioning. However, the Bat system is based on wired networks, so the large-scale deployment could be restricted by costs.

The Cricket Positioning System developed by the MIT Artificial Intelligence Lab, specifically aimed at indoor environment applications [10,11], consists of beacons permanently placed in the buildings, nodes equipped on the target and a central server. The ID of every beacon is unique in order to identify its position coordinates and to enable the broadcasting of an RF signal with its self-location information. The objective nodes launch an ultrasonic signal in response to the RF signal. After obtaining the response from beacons, the distances between the beacons are able to be calculated by the TDOA method. The position information can be figured out based on the relevant localization algorithm.

The SUPPER-ID(S-ID) system [12] uses infrared distance measurements to assist ultrasonic distance measurements and to realize position estimation by means of the trilateral positioning principle. The system can solve the deficiencies caused by employing ultrasonic wave or infrared distance measurement independently and extends the coverage range of a single node so as to improve localization accuracy.

All of the above systems are typical three-dimensional positioning systems. Though they can guarantee the proper accuracy of three-dimensional localization, each of them has weaknesses, such as relatively small coverage area, high cost and the tedious manual deployment of large quantities of beacons, which result in the unavailability of self-localization for nodes. However, the distance measurement manners and the hardware system design for measurements are still significant to the three-dimensional positioning research based on WSNs.

In addition, in terms of the two-dimensional WSN positioning algorithm research, many methods have been proposed, which can be classified into two main clusters [13]: range-based and range-free localization algorithms. The former estimates the node’s location by measuring the distance and angle information between each node. The latter realizes localization depending on the network connectivity. The range-based algorithm mainly depends on the following measurements: received signal strength (RSS) [14], angle of arrival (AOA) [15], time of arrival (TOA) [16] and time difference of arrival (TDOA) [17]. The range-free technique algorithm mainly includes: a centroid localization algorithm [18], convex programming [19], DV-hop [20], DV-distance [21], MDS-MAP [22], APIT [23], and so on.

The investigation of WSN positioning techniques in the two-dimensional space is relatively mature. However, the majority of the algorithms cannot fit the localization well in 3D settings. At present, the localization study with respect to 3D scene primarily modifies the classical two-dimensional ones to extend to one more dimension. The main idea is to use spatial geometrical relations, such as spherical coordinates, hyperboloid coordinates, sphere segmentation and cube segmentation, to divide the scene into possible spaces where unknown nodes stay. The centroid of the possible space is the estimation of the unknown node’s coordinates. Quadrilateration and maximum likelihood estimation can also be utilized to determine the coordinates of unknown nodes directly. The literature [24] proposes a 3D self-localization method based on WSNs, named the APIT-3D algorithm, which improves the APIT algorithm to adapt to 3D space. The work in [25] presents a distributed three-dimensional centroid localization algorithm on the basis of the centroid algorithm. The work in [26] advances three two-dimensional positioning algorithms, respectively, and comes up with the 3D-Dv-hop, 3D-centroid and 3D-Dv-distance algorithms [27]. The literature [28] proposes the 3D-MDS algorithm after extending the MDS-MAP to 3D and presents the self-localization method for nodes of WSNs, solving the self-localization problem of the unattended nodes located at unknown places [29].

In 3D Wi-Fi-based localization, most Wi-Fi-based solutions require a process of site survey, where Wi-Fi signatures of an area of interest are annotated with their real recorded locations [30]. The Wi-Fi signatures, *i.e.*, the fingerprint, commonly consist of RSSIs from different APs (access points) in a database after a site survey. When the current fingerprint matches one in the database, the location of interest can be located by the position information corresponding to the fingerprint [31]. Generally the Wi-Fi-based localization accuracy is only able to reach the meter level, mainly because RSSI is highly vulnerable to the effects of furniture and other objects in a room. The density of APs is also sparse, which does not ensure high granularity of localization information [32]. However, in 3D WSN localization, the distance between two nodes can be measured by TOA or TDOA, instead of RSSI, and the density of nodes can be customized according to the application requirements. Therefore, WSN-based localization can reach centimeter level precision in indoor settings. In addition, a site survey, which is necessary for Wi-Fi-based localization, is time and labor intensive. In short, WSNs have much higher localization accuracy than Wi-Fi in indoor circumstances.

## 3. System Overview

**Figure 1:**
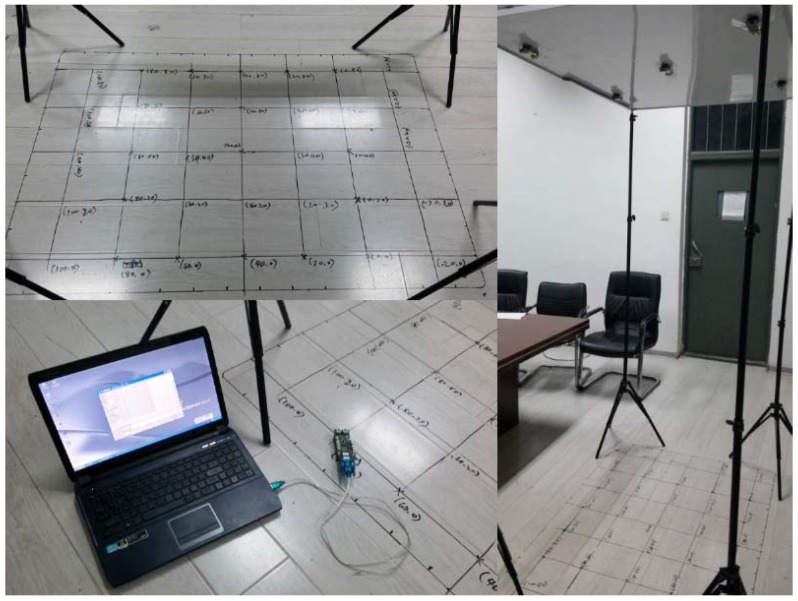
The deployment of the system.

All of the following research is studied in indoor environments. Compared to outdoor WSNs, indoor WSNs are more likely to be customized for the particular application, like locating or tracking an object of interest. In indoor scenes, the primary consideration is how to deploy sensor nodes in 3D space to guarantee accurate communication among nodes. Inspired by ancient navigation, we stick the sensor nodes to the ceiling of the room, like the Sun or constellations, which ancient sailors observed using a sextant or some other instruments to locate the boat. The deployment is shown in Figure 1, where the nodes on the ceiling are beacons, and the node equipped with the moving target is the listener. Furthermore, the Cricket system is employed in a real experiment, and the deployment strategy is capable of ensuring ultrasonic transmission between any two nodes that are in the line of sight (LOS) to the greatest extent.

**Figure 2:**
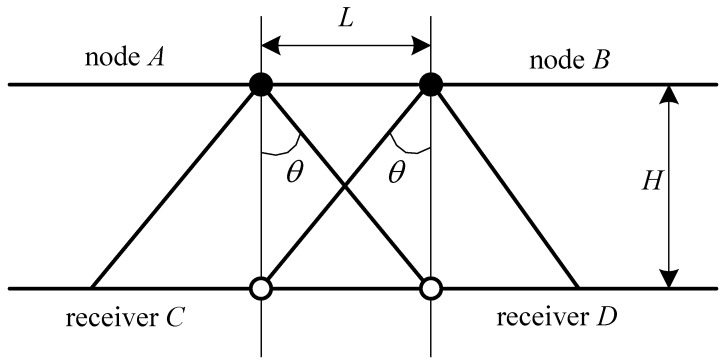
The side view of the node layout.

The layout of beacons on the ceiling should be discussed in terms of the number and locations of beacons. Since the transmitting angle of an ultrasonic sensor equipped in the node has an effective transmitting range, the layout of beacons should guarantee that the joint transmitting range is able to cover the monitoring field as much as possible. In the real experiment, we deploy five beacons on the ceiling, which are shown in Figure 1. According to the empirical measurement, the effective transmitting range of an ultrasonic sensor is a cone with about a 60-degree cone angle. Assuming half of the cone angle is *θ*, tanθ is approximate to 2/3 for simplifying the calculation. The side view of the node layout is shown in Figure 2, where tanθ=L/H, and H=209.0 cm and L=139.3 cm in our experiment.

For a bigger ultrasonic coverage range, the distance between any two of five beacons should be less than *L*. Through practical measurement, the 3D coordinates of the five beacons are (0,0,209), (80,0,209), (0,80,209), (80,80,209) and (40,40,209), respectively, and the maximum distance between nodes is 113 cm, which fits the layout requirement.

Based on the experimental platform shown in Figure 1, we evaluate our methods mentioned in the following sections to locate and track the unknown target. After adequate simulation and experiments, we are able to effectively shrink the localization error into centimeter-level precision, and the accuracy can already satisfy most indoor localization-oriented applications.

## 4. The Self-Localization of Beacons

Many self-localization methods have been proposed to determine nodes’ coordinates by varying researchers. The majority of existing works is designed for specific application backgrounds; thus, we present here a self-localization scheme suitable for an indoor Cricket-like system, which deploys all nodes, including beacons, onto the ceiling. As described in the previous section, the beacons and the target are not in the same plane. Based on the layout of beacons, it is known that all of the beacons should be in the same plane, which means their z-axes are also identical, meanwhile the ground on which targets move should be parallel to the ceiling on which the nodes are mounted. Under these assumptions, the goal of the self-localization is to determine the accurate locations of the beacons.

Aimed at the research background, we apply the Cayley–Menger determinant to calculate the coordinates of beacons. The Cayley–Menger determinant is always used in distance geometry for determining the volume of a triangular pyramid based on the distances between any two of four vertices. For instance, there is a triangular pyramid with four vertices $p_{1}$, $p_{2}$, $p_{3}$ and $p_{4}$, and the relation between the volume of the triangular pyramid and its Cayley–Menger determinant can be formulated as $36V^{2}=D\left(p_{1},p_{2},p_{3},p_{4}\right)$, where *V* is the volume of the triangular pyramid, and D(·) indicates the Cayley–Menger determinant, the expression of which is denoted as:

(1) $$D\left(p_{1},\ldots ,p_{n},q_{1},\ldots ,q_{n}\right)=2{\left(-\frac{1}{2}\right)}^{n}\left|\begin{array}{cccc} 0 & 1 & \cdots & 1\\ 1 & D(p_{1},q_{1}) & \cdots & D(p_{1},q_{n})\\ \vdots & \vdots & \ddots & \vdots \\ 1 & D(p_{n},q_{1}) & \cdots & D(p_{n},q_{n}) \end{array}\right|$$

where $D(p_{i},q_{j})$ is the square of the Euclidean distance between $p_{i}$ and $q_{j}$; while $D(p_{1},p_{2},p_{3},p_{4})$ is the short form of $D\left(p_{1},p_{2},p_{3},p_{4},q_{1},q_{2},q_{3},q_{4}\right)\;\left(p_{i}=q_{j},i=j=1,2,3,4\right)$. Thus, the linear relation between volume and edge lengths, or rather coordinates of vertices, of the triangular pyramid is built by the Cayley–Menger determinant.

**Figure 3:**
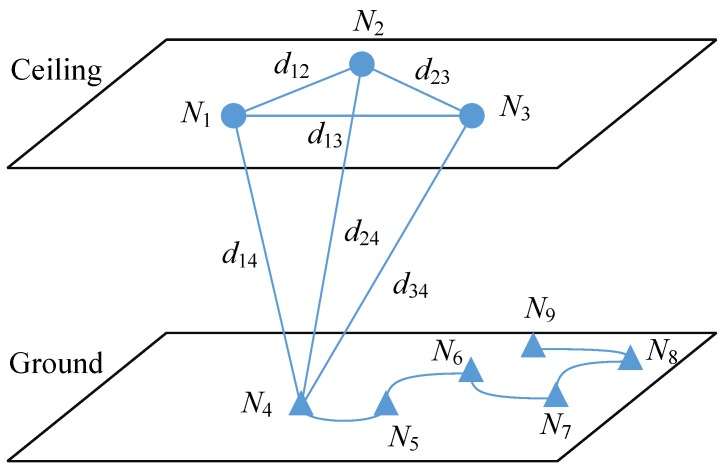
The illustration of the 3D self-localization system.

The illustration of the 3D self-localization system is shown in Figure 3, where the circle points $N_{i}\;\left(i=1,2,3\right)$ refer to beacons and the triangle points $N_{i}\;\left(i=4,\ldots ,9\right)$ refer to the locations through which the target moves. Using ultrasound measurements, the set of distances $D_{j}=\left\{d_{1j},d_{2j},d_{3j}\right\}\;$(j=4,…,9) can be acquired as targets move via the trajectory from $N_{4}$ to $N_{9}$. Due to the assumption that the ceiling is parallel to the ground, the volumes $V_{123j}\;\left(j=4,\ldots ,9\right)$ of the triangular pyramids, one of which consists of three beacons, and one target measurement location are identical. According to Equation (1), we can obtain the following result:

(2) $$288V_{123j}^{2}=\left|\begin{array}{ccccc} 0 & 1 & 1 & 1 & 1\\ 1 & 0 & d_{12}^{2} & d_{13}^{2} & d_{1j}^{2}\\ 1 & d_{12}^{2} & 0 & d_{23}^{2} & d_{2j}^{2}\\ 1 & d_{13}^{2} & d_{23}^{2} & 0 & d_{3j}^{2}\\ 1 & d_{1j}^{2} & d_{2j}^{2} & d_{3j}^{2} & 0 \end{array}\right|$$

where $d_{ij}$ is the Euclidean distance between $N_{i}$ and $N_{j}$.

After expanding the determinant in Equation (2), an equation set can be expressed as:

(3) AX=B

where:

$$A=\left[\begin{array}{cccccc} d_{34}^{2} & d_{24}^{2} & d_{14}^{2} & D_{324}D_{214} & -D_{314}D_{214} & -1\\ d_{35}^{2} & d_{25}^{2} & d_{15}^{2} & D_{325}D_{215} & -D_{315}D_{215} & -1\\ \vdots & \vdots & \vdots & \vdots & \vdots & \vdots \\ d_{39}^{2} & d_{29}^{2} & d_{19}^{2} & D_{329}D_{219} & -D_{319}D_{219} & -1 \end{array}\right]$$

$$X=\frac{1}{d_{12}^{2}}\left[\begin{array}{c} d_{12}^{2}\left(d_{13}^{2}+d_{23}^{2}-d_{12}^{2}\right)\\ d_{13}^{2}\left(d_{12}^{2}+d_{23}^{2}-d_{13}^{2}\right)\\ d_{23}^{2}\left(d_{12}^{2}+d_{13}^{2}-d_{23}^{2}\right)\\ d_{13}^{2}\\ d_{23}^{2}\\ 144V_{t}^{2}+d_{12}^{2}d_{13}^{2}d_{23}^{2} \end{array}\right]$$

$$B=\left[\begin{array}{c} D_{314}D_{324}\\ D_{315}D_{325}\\ \vdots \\ D_{319}D_{329} \end{array}\right]$$

$$D_{ijk}=d_{ik}^{2}-d_{jk}^{2}\;\left(i,j=1,2,3;k=4,5,6,\ldots ,9\right)$$

All of the elements of *A* and *B* are measurable by time of arrival (TOA), which has centimeter-level accuracy in indoor environments. The vector of *X* is composed of $d_{12}$, $d_{13}$ and $d_{23}$, which are unknown variables that need to be determined. Let $A^{\prime }$ and $B^{\prime }$ be the practical measurement matrices of *A* and *B*, which involve measurement errors. Then, the least squares estimator of *X* is as follows:

(4) $${\hat{X}}_{LS}={\left(A^{\prime T}\cdot A^{\prime }\right)}^{-1}A^{\prime T}\cdot B^{\prime }$$

where ${\hat{X}}_{LS}$ is a five-dimensional vector in the example, which is indicated as $\left[X_{1},X_{2},X_{3},X_{4},X_{5}\right]$. Thus:

(5) $$\begin{array}{cc} d_{12} & =\frac{1}{\sqrt{2}}\sqrt{\frac{X_{2}}{X_{4}}+\frac{X_{3}}{X_{5}}}\\ d_{13} & =\frac{1}{\sqrt{2}}\sqrt{X_{1}+\frac{X_{3}}{X_{5}}}\\ d_{23} & =\frac{1}{\sqrt{2}}\sqrt{X_{1}+\frac{X_{2}}{X_{4}}} \end{array}$$

We can estimate the coordinates of beacons by Equation (5) as:

(6) $$\begin{array}{cc} N_{1} & =(0,0)\\ N_{2} & =(d_{12},0)\\ N_{3} & =\left(\frac{d_{12}^{2}+d_{13}^{2}-d_{23}^{2}}{2d_{12}},\pm \sqrt{d_{13}^{2}-\left(\frac{d_{12}^{2}+d_{13}^{2}-d_{23}^{2}}{2d_{12}}\right)}\right) \end{array}$$

When the number of beacons is over three, any three of them could determine a coordinate system, and all of the varying coordinate systems can be unified by corresponding transformation matrices, which can be easily obtained. Given the number of beacons is five, $N_{4}$ and $N_{5}$ are as follow:

(7) $$\begin{array}{cc} N_{4} & =\left(\frac{d_{12}^{2}+d_{14}^{2}-d_{24}^{2}}{2d_{12}},\pm \sqrt{d_{14}^{2}-\left(\frac{d_{12}^{2}+d_{14}^{2}-d_{24}^{2}}{2d_{12}}\right)}\right)\\ N_{5} & =\left(\frac{d_{12}^{2}+d_{15}^{2}-d_{25}^{2}}{2d_{12}},\pm \sqrt{d_{15}^{2}-\left(\frac{d_{12}^{2}+d_{15}^{2}-d_{25}^{2}}{2d_{12}}\right)}\right) \end{array}$$

Since measurement errors always have negative influences on estimated consequences, differential sensitivity analysis (DSA) is employed to eliminate the influence in this paper. DSA is a method to approximate the function’s variance by first order Taylor series for estimating the function deviation derived from arguments with noise.

The vector $Y=[y_{1},\ldots ,y_{l}]$ includes *l* variables, which are $y_{u}=g_{u}\left(Z\right)\;\left(u=1,\ldots ,l\right)$. Suppose that there is a set $\{z_{1},z_{2},\ldots ,z_{p}\}\in Z$ in which every element has noise and follows a normal distribution of mean $\bar{z_{k}}$ and variance $\sigma_{z_{k}}^{2}$. Then, the covariance Cov(Y) can be expressed as:

(8) $$Cov\left(Y\right)=RCov\left(Z\right)R^{T}$$

where Cov(·) denotes the covariance function and *R* is the Jacobian matrix $G=\{g_{1},g_{2},\ldots ,g_{l}\}$ of *Z*.

Based on DSA, Equation (3) can be converted to:

(9) $$A^{\prime }X+H=B^{\prime }$$

where *H* is the residual error vector. Additionally, the covariance matrix Cov(H) is able to be presented as:

(10) $$Cov\left(H\right)=Cov\left(B^{\prime }-A^{\prime }X\right)=RCov\left(D^{M}\right)R^{T}=\sigma_{M}^{2}RR^{T}$$

where $D^{M}$ refers to the set of all of the measured distances and *R* refers to the Jacobian matrix of G=B−AX with respect to $D^{M}$. To simplify this case, we assume that all of the measured distances have the same variance $\sigma_{M}^{2}$.

To minimize the sum of square of weighted errors:

$$J_{W(\hat{x})}={\left(B^{\prime }-A^{\prime }\hat{X}\right)}^{T}W\left(B^{\prime }-A^{\prime }\hat{X}\right)$$

where $W=diag(w_{1},w_{2},\cdots ,w_{n})$, we can acquire the weighted least squares (WLS) estimation $\hat{X_{WLS}}$ as:

(11) $$\hat{X_{WLS}}={\left(A^{\prime T}Cov{\left(H\right)}^{-1}A^{\prime }\right)}^{-1}A^{\prime T}Cov{\left(H\right)}^{-1}B^{\prime }$$

Note that the Cov(H) could be calculated by *X* with noise, thus *X* will be replaced by ${\hat{X}}_{LS}$ in the real calculation.

## 5. The Calibration of Beacons

### 5.1. Dead Reckoning

The previous section provided a feasible self-localization method that can position unknown beacons by measuring the distances between beacons and the target. Though the measurement error has been taken into account in the method, it is necessary to calibrate the position of the beacons. In the present paper, we utilized a moving robot with odometers to calibrate the locations of beacons as the robot is moving in the area covered by WSNs.

The moving robot UP-voyager II, which is used as the target in our experiment, is driven by the differential actuator mode. This means that there are only two motor-driven wheels with optical-electricity encoders on the robot, and the moving trajectory of the robot can be controlled by forwarding and reversing the wheels. To build the robot’s motion model, we assume that the robot is regarded as a mass point, and it merely moves on a 2D plane. The robot’s pose includes its position and orientation and can be expressed as the vector $q_{k}={\left(x_{k},y_{k},\theta_{k}\right)}^{T}$, where $\left(x_{k},y_{k}\right)$ indicates the robot’s coordinate at time *k*, and $\theta_{k}$ indicates the robot’s orientation, *i.e.*, the angle between the velocity direction and positive x-axis, at time *k*. Let the radii of the two wheels be $R_{l}$ and $R_{r}$, respectively, and the spacing distance of them be *a*. The optical-electricity encoder has *P* slits/rad and outputs *N* impulses in unit interval Δt. Thus, the rotation distances of the two wheels are presented respectively as:

(12) $$\begin{array}{cc} \Delta d_{l} & =2*\frac{N}{P}*\pi *R_{l}\\ \Delta d_{r} & =2*\frac{N}{P}*\pi *R_{r} \end{array}$$

**Figure 4:**
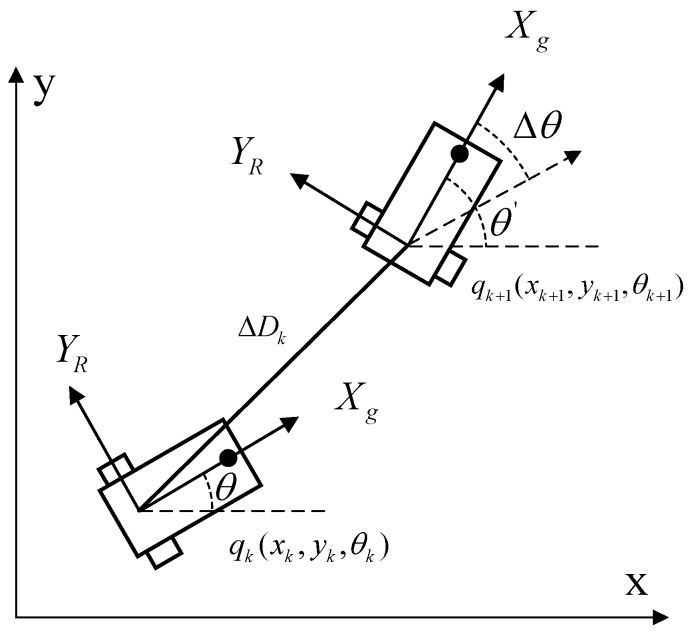
The motion model of the moving robot.

Based on dead reckoning, the illustration of the robot moving from the present state to the next state is shown in Figure 4, where $q_{k+1}={\left(x_{k+1},y_{k+1},\theta_{k+1}\right)}^{T}$ refers to the robot’s pose at the next time step k+1, $\Delta D_{k}=\left(\Delta d_{l}+\Delta d_{r}\right)/2$ refers to the moving distance during unit time Δt and $\Delta \theta_{k}=\left(\Delta d_{l}-\Delta d_{r}\right)/a$ refers to the variation of the robot’s orientation. Let $u_{k}={\left[\Delta D_{k},\Delta \theta_{k}\right]}^{T}$ be input information at time step *k*; then, the motion model of the robot can be expressed as

(13) $$q_{k+1}=f\left(q_{k},u_{k}\right)+w_{k}$$

where:

$$f\left(q_{k},u_{k}\right)=\left[\begin{array}{c} x_{k}+\Delta D_{k}cos\left(\theta_{k}+\Delta \theta_{k}/2\right)\\ y_{k}+\Delta D_{k}sin\left(\theta_{k}+\Delta \theta_{k}/2\right)\\ \theta_{k}+\Delta \theta_{k} \end{array}\right]$$

and $w_{k}$ is the noise of the encoder. Due to the assumption that the robot simply moves on a 2D plane, the Jacobian matrix of $f(q_{k},u_{k})$ with respect to $q_{k}$ is shown as:

(14) $$A_{k+1}=\frac{\partial f}{\partial q}{|}_{q=q_{k}}=\left[\begin{array}{ccc} 1 & 0 & -\Delta D_{k}sin\left(\theta_{k}+\Delta \theta /2\right)\\ 0 & 1 & \Delta D_{k}cos\left(\theta_{k}+\Delta \theta /2\right)\\ 0 & 0 & 1 \end{array}\right]$$

If the sampling interval is small enough, the robot’s motion model, shown as Equation (13), could match the real trajectory extremely well.

#### The Cricket System

We suppose that the coordinates of beacon *i* are $\left(bx_{i},by_{i},bz_{i}\right)$, and the state of the robot is $q_{k+1}$; thus, the distance between them at time step k+1 is:

$$d_{k+1}=\sqrt{{\left(x_{k+1}-bx_{i}\right)}^{2}+{\left(y_{k+1}-by_{i}\right)}^{2}+{\left(z_{k+1}-bz_{i}\right)}^{2}}+v_{k}$$

where $v_{k}$ is the measurement noise being subject to a normal distribution with mean zero.

**Figure 5:**
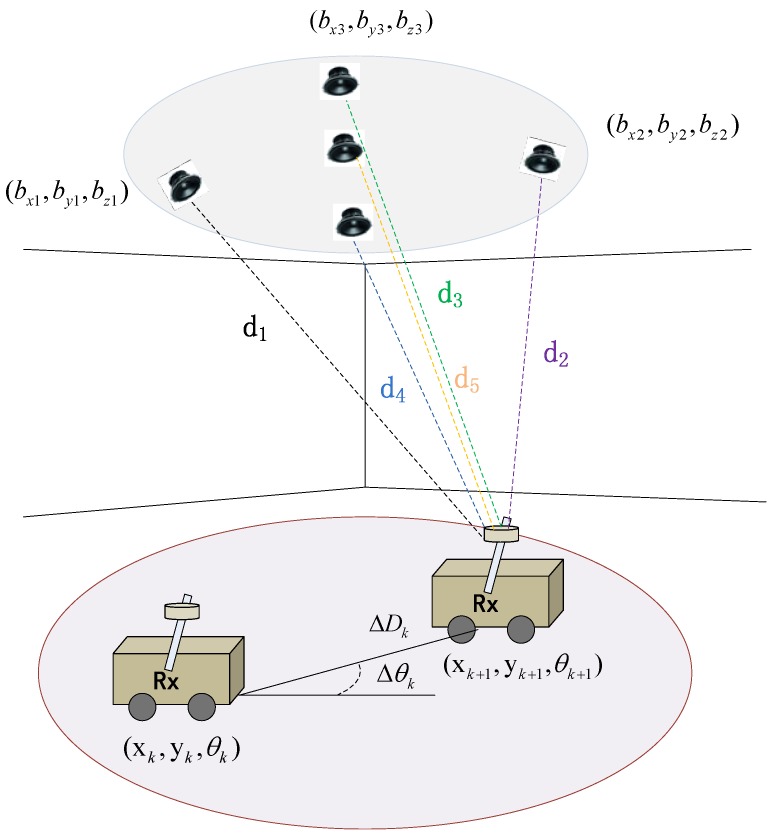
The Cricket system.

How the Cricket system works is briefly illustrated in Figure 5. For calibrating beacons, the state $q_{k}$ should be extended to $q_{k}={\left[x_{k},y_{k},\theta_{k},bx_{1},by_{1},bz_{1},\ldots ,bx_{n},by_{n},bz_{n}\right]}^{T}$, and $q_{k+1}$ can be derived from $q_{k}$ based on Equation (13), which is shown as:

(15) $$q_{k+1}=\left[\begin{array}{c} x_{k}+\Delta D_{k}cos\left(\theta_{k}+\Delta \theta /2\right)\\ y_{k}+\Delta D_{k}sin\left(\theta_{k}+\Delta \theta /2\right)\\ \theta_{k}+\Delta \theta_{k}\\ bx_{1}\\ \vdots \\ bz_{n} \end{array}\right]+w_{k}$$

To simplify the calculation, Equation (15) can be linearized to be:

(16) $$\begin{array}{cc} q_{k+1} & =Aq_{k}+w_{k}\\ d_{k+1} & =Cq_{k+1}+v_{k} \end{array}$$

where *A* and *C* are the Jacobian matrices of state matrix and measurement matrix, respectively, $w_{k}$ and $v_{k}$ follow a normal distribution with the same mean zero and different variances, *Q* and *R*, respectively.

### 5.2. Location Optimization Based on Filter Methods

The traditional filter methods, like the Kalman filter, require accurate statistical features of a system’s model and noise to be known, so does the extended Kalman filter (EKF). The EKF, the nonlinear version of the Kalman filter, linearizes nonlinear functions by first order Taylor series to approximate the nonlinear system [33]. However, the noise of the model and measurement cannot be avoided in a practical experiment. The $H_{\infty }$ filter is the filter method proposed for the system with an uncertain model and noise distribution, and it is verified to have robust performance in such a case. The $H_{\infty }$ filter is also called the minimax filter, which can minimize the maximum estimation error; thus, it is allowed to estimate a state with unknown or hardly determinate noise features [34]. The $H_{\infty }$ filter just assumes that the noise is the energy-limited signal, which accords more perfectly with the practical application situation. The literature [35] concludes that compared to the Kalman filter, the $H_{\infty }$ filter is less sensitive to the change of variances.

The state and measurement model are presented as Equation (16). Let $\hat{q_{k}}$ be the system state estimator; the goal of the $H_{\infty }$ filter is to satisfy the following condition:

(17) $$\min_{\hat{q_{k}}}\max_{w_{k},v_{k}}J$$

where *J* is the assessment function for the filter performance. Considering the worst situation of $w_{k}$ and $v_{k}$, *i.e.*, the worst influence on the estimator from the noise, the function of *J* can be defined as:

(18) $$J=\frac{\sum_{k=0}^{N-1}{∥q_{k}-\hat{q_{k}}∥}_{Q}^{2}}{\sum_{k=0}^{N-1}∥w_{k}{∥}_{W}^{2}+\sum_{k=0}^{N-1}{∥v_{k}∥}_{V}^{2}}$$

where W>0, V<0 and Q>0 are weighted matrices.

To obtain the optimal estimator $\hat{q_{k}}$, *J* should meet the condition of J<1/γ, and *γ* is the scalar designated by users, which can be regarded as the presupposed noise attenuation.

The recursion process of the $H_{\infty }$ filter is as follows:

(19) $$\begin{array}{cc} L_{k} & ={\left(I-\gamma MP_{k}+C^{T}R^{-1}CP_{k}\right)}^{-1}\\ K_{k} & =AP_{k}L_{k}C^{T}R^{-1}\\ {\hat{q}}_{k+1} & ={\hat{q}}_{k+1,odo}+K_{k}\left(d_{k+1}-C{\hat{q}}_{k+1,odo}\right)\\ P_{k+1} & =AP_{k}L_{k}A^{T}+Q \end{array}$$

where ${\hat{q}}_{k+1,odo}$ is the optimal estimator based on the odometer data at the last time step, $K_{k}$ is the gain factor of the filter and γM is the parameter of the filter. According to Equation (19), it is easy to find that the $H_{\infty }$ filter is similar to the Kalman filter to some extent. When γ→0, the $H_{\infty }$ filter is an extremely close approximation to the Kalman filter, with the minimum variance for the estimator; when γ→∞, the $H_{\infty }$ filter has the most robustness. Therefore, appropriately choosing the value of *γ* facilitates a tradeoff between the robustness of the filter system and estimation variance.

Besides the $H_{\infty }$ filter, we utilize another filter named the strong tracking filter (STF) to refine estimators, because it is the improved version of the extended Kalman filter to solve the filter problem of a nonlinear system. Compared to the Kalman filter, STF is one of the improved Kalman filter methods: it orthogonalizes the residual error series at every step to extract the useful information from the residual error to estimate the current state [36]. Thus, STF has more robust performance against the mismatch of the model’s parameters than the Kalman filter.

To summarize, the key point of STF is to select an appropriate time-variant gain matrix K(k+1) to make the following equation true.

(20) $$E[\gamma \left(k+1+j\right)\gamma^{T}\left(K+1\right)]=0\;\left(k=0,1,2,\ldots ,j=1,2,3,\ldots \right)$$

where γ(k+1) is the residual error vector of the measurement matrix, and the residual error sequences in varying time steps should maintain an orthogonality relationship, which is formulated in Equation (20).

When the state model exactly matches the real situation, the residual error of the output of the Kalman filter is a series of non-autocorrelation white noise, which satisfies Equation (20). However, under the influence of the model’s uncertainty, the disturbances on the mean and amplitude of the outputs of the residual error sequences are inevitable; the gain matrix needs to be modified to make Equation (20) still true. Forcing STF to maintain tracking of the real system state is the attribute of STF.

We evaluate the $H_{\infty }$ and STF algorithms via simulation, and the details are shown in the following section. For simplicity, we use HF to stand for the $H_{\infty }$ filter from now on.

## 6. The Localization of Targets

The last two sections provided the corresponding methods to locate and calibrate the beacons’ positions. To realize the localization of targets, we propose to respectively apply the Gauss–Newton iterative method and the Cayley–Menger determinant. As only three beacons receive the returned ultrasonic signal, an equation set F(X) of three equations can be established by signal measurements, but three equations are not enough to limit any non-linear part. Thus, the Gauss–Newton iterative method and the Cayley–Menger determinant are mainly used to dispel the non-linear part for estimating the solution of equations, *i.e.*, the location of the target. The two methods will be evaluated via the experiment in the following section.

This section principally gives the optimal node selection scheme based on geometric dilution precision (GDOP). In GPS, the localization accuracy could be influenced by the deployment of beacons and the target, and the same phenomenon also happens in the localization of WSNs [37]. Since GDOP can reflect the scaling degree of measurement error, the optimal nodes are the ones that have the minimum GDOP. Based on the definition, GDOP is the amplification coefficient from measurement error to localization error, which is as:

(21) $$\begin{array}{cc} GDOP & =\sqrt{\frac{E\left[\Delta x^{2}\right]+E\left[\Delta y^{2}\right]+E\left[\Delta z^{2}\right]}{E[\Delta \rho^{2}]}}\\ & =\sqrt{\frac{\sigma_{x}^{2}+\sigma_{y}^{2}+\sigma_{z}^{2}}{\sigma_{\rho }^{2}}} \end{array}$$

where $\sigma_{\rho }$ is the vector of measurement error and $\sigma_{X}=\left[\sigma_{x},\sigma_{y},\sigma_{z}\right]$ is the vector of localization error. We suppose that the variances of all of the measurement distances of beacons are identical, $\sigma_{\rho }^{2}$, then the covariance of $\sigma_{X}$ will be obtained:

(22) $$C\left(\sigma_{X}\right)=\left[\begin{array}{ccc} \sigma_{x}^{2} & & \\ & \sigma_{y}^{2} & \\ & & \sigma_{z}^{2} \end{array}\right]=\sigma_{\rho }^{2}{\left(J^{T}J\right)}^{-1}$$

where *J* is the Jacobian matrix of F(X). Let *G* be:

(23) $$G={\left(J^{T}J\right)}^{-1}=\left[\begin{array}{ccc} G_{11} & G_{12} & G_{13}\\ G_{21} & G_{22} & G_{23}\\ G_{31} & G_{32} & G_{33} \end{array}\right]$$

The GDOP in the localization system can be derived by Equations (22) and (23) as:

(24) $$GDOP=\sqrt{trace(G)}=\sqrt{G_{11}+G_{22}+G_{33}}$$

Generally, the number of beacons is greater than three, so a method of picking the combination of three beacons that minimizes GDOP needs to be discussed. Intuitively, the number of combinations is $C_{N}^{3}$, as there are *N* beacons deployed. The strategy is to find the combination that has the minimum GDOP in all. Based on the strategy, we calculate the GDOP of all of the beacons, which is shown as follows.

**Figure 6:**
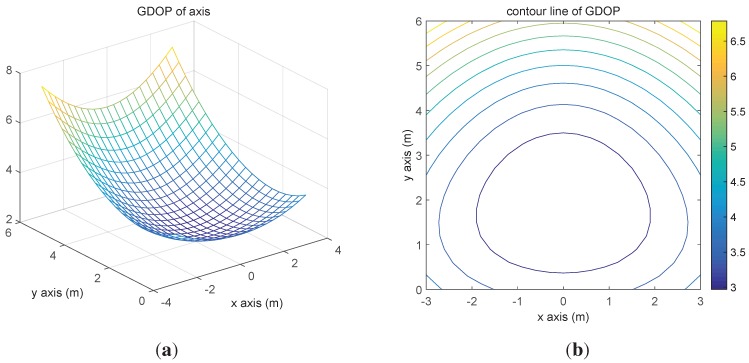
Geometric dilution precision (GDOP) distribution. (**a**) GDOP of the axis; (**b**) contour line of GDOP.

The GDOPs of every node are plotted in Figure 6a, and the corresponding contour line is shown in Figure 6b. According to the two figures, it can be concluded that picking beacons in the middle of the deployment could provide a more accurate location of the target than others.

In practice, we can also apply the measurement information from other beacons besides the three picked ones, which means more information will be merged into the three picked ones. Thus, the GDOP-based weighting estimation method (GWEM) is proposed in the paper to realize information fusion. For simplicity and clearness, the method will be introduced by steps.

- Assume that there are *N* beacons in the experiment, which can compose *M* combinations where $M=\sum C_{N}^{i}$ (i=3), and all of the combinations’ index set can be expressed as $S_{k}|k=1,2,\ldots ,M$
- To every combination, employ the Cayley–Menger determinant to estimate the location of the target and obtain the estimated consequence $\hat{X_{k}}$, then the corresponding $GDOP(\hat{X_{k}},S_{k})$ can be derived.
- Weight the sum of the consequences of every combination as the following equation:
  (25) $$\hat{X}=\frac{\sum_{k=1}^{M}\hat{X_{k}}{\left(GDOP^{2}\left(\hat{X_{k}},S_{k}\right)\right)}^{-1}}{\sum_{k=1}^{M}{\left(GDOP^{2}\left(\hat{X_{k}},S_{k}\right)\right)}^{-1}}$$

## 7. Experiment

**Figure 7:**
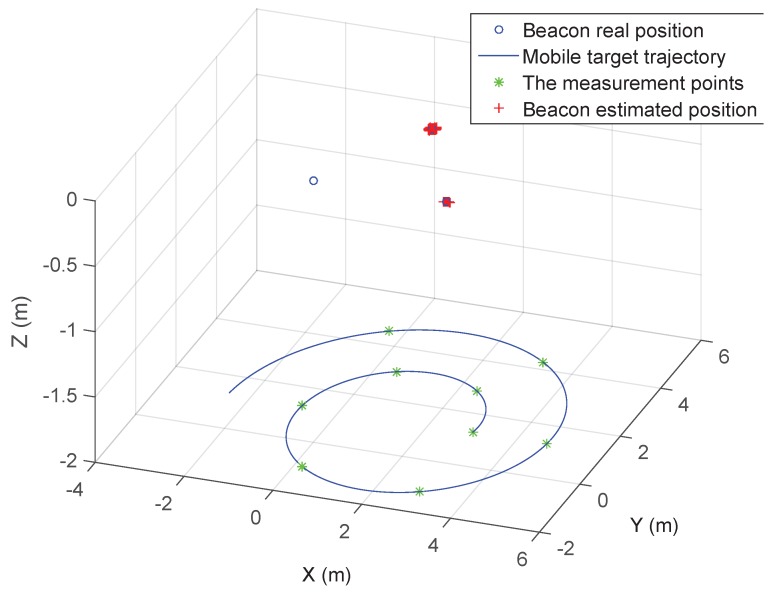
The simulation configuration.

To evaluate the proposed algorithms, we design and conduct a series of simulations and experiments, and the setting of the experiment is described as the statement in the System Overview section. Firstly, we design the simulation to verify the algorithm for the self-localization of beacons, the configuration of which is illustrated in Figure 7. In the figure, we assume that there are three beacons represented by a blue circle on the ceiling, the coordinates of which are [0,0,0], [3,0,0] and [1.5,2.6,0] respectively.

In the meantime, the mobile target moves along the spiral trajectory, which can be represented as:

(26) $$l=R_{0}+\frac{3(R_{M}-R_{0})}{\pi m}\theta$$

where $R_{0}=1.5$ m, $R_{M}=4$ m and m=9. The spiral trajectory can guarantee that the measurement information is sufficient for algorithm processing, and the singular point could be avoided efficiently [38]. The measurement points, shown by green stars, are deployed along the trajectory every 60 degrees, and it is assumed that the measurement noise is also white noise with standard variance σ=0.02 m. The red cross refers to the position estimated by the proposed self-localization algorithm.

**Figure 8:**
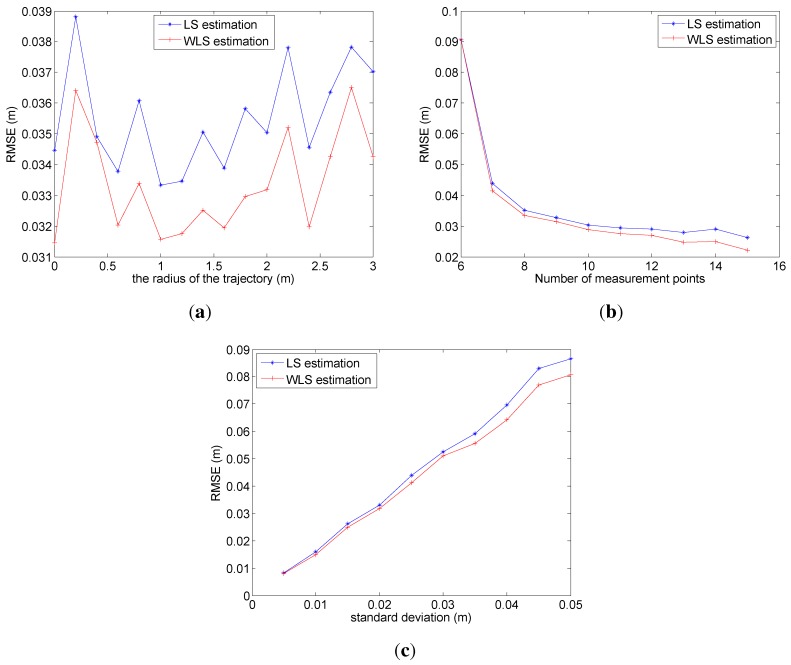
The illustration of impacts. (**a**) The impact of the radius of the spiral trajectory; (**b**) the impact of the number of measurement points; (**c**) the impact of environment noise.

To further analyze the adaptiveness and robustness of the algorithm, the following simulation is conducted. For surveying the impact of the radius of the spiral trajectory on localization accuracy, the varying original radii are picked for the simulation, and the corresponding average RMSE of localization by 100 times of simulation is represented in Figure 8a. In Figure 8a, it is easy to find out that the optimal original radius is 1 m regardless of LS or WLS, and the WLS method is more robust than the LS with the variation of the radius.

In addition, the impact of the number of measurement points on localization accuracy is shown in Figure 8b. Obviously, following the increase of the number, WLS has a better performance than LS. When the number is over 12, the trend of RMSE by LS begins to flatten, while the one of WLS still descends, which demonstrates that WLS can apply redundant measurements more efficiently than LS.

Then, the impact of environment noise on localization accuracy is considered and shown in Figure 8c. In Figure 8c, the RMSE increases with an approximate linear growth as the standard deviation of environment noise rises, and the slope of the approximate linear relationship is about 1.4. Under the same conditions of simulation, WLS has 10% higher localization accuracy than LS.

**Figure 9:**
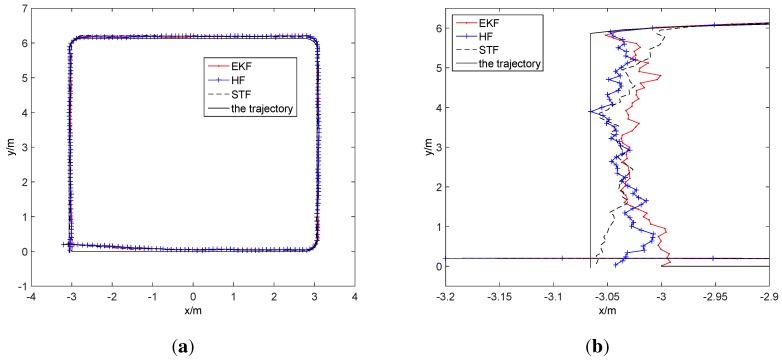
The tracking by filters. (**a**) The performance comparison; (**b**) the partial enlarged view.

Secondly, we redeploy up to five beacons and redesign the trajectory of the mobile target in the simulation to evaluate the calibration effect by the proposed scheme. The five beacons are deployed at the coordinates of [0,3,3], [−2,1,3], [2,1,3], [−2,5,3] and [2,5,3], respectively. Let the sampling interval be 1 s, the standard deviation of the noise of the distance measurement be 3 cm and the standard deviation of the noise of the odometer be 1 cm. The starting point of the target is at [−3,0,0], and the target moves along a square with a 6-m side length. Under the above conditions, the EKF, HF and STF algorithms are applied to track the trajectory, and the result of tracking is shown in Figure 9.

**Figure 10:**
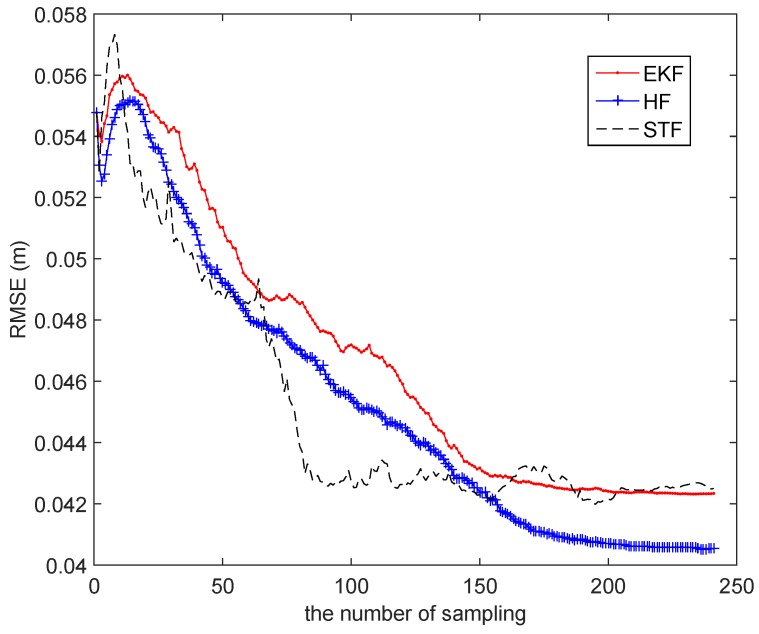
The RMSE of EKF, the $H_{\infty }$ filter (HF) and the strong tracking filter (STF).

In Figure 9, the three filters all provide highly accurate tracking results. For surveying the consequences subtly, the left part of Figure 9a is enlarged to Figure 9b, and in Figure 9b, it is shown that HF and STF have a better performance than EKF. The average localization error of EKF is [0.0336 m, 0.0644 m, 0.0244 (rad)], the one of HF is [0.0287 m, 0.0612 m, 0.0233 (rad)] and the one of STF is [0.0338 m, 0.0669 m, 0.0221 (rad)]. Following the number of sampling increases, the RMSEs of the three approaches obviously have a downward trend, which is shown in Figure 10.

In Figure 10, it is presented that the RMSE of STF falls with the fastest speed, and it is the first one to reach a stable state in the three schemes. Thus, STF can satisfy tracking in the shortest possible time, while HF has the best overall effect compared to the two others and demonstrates the excellent adaptiveness of the models.

**Figure 11:**
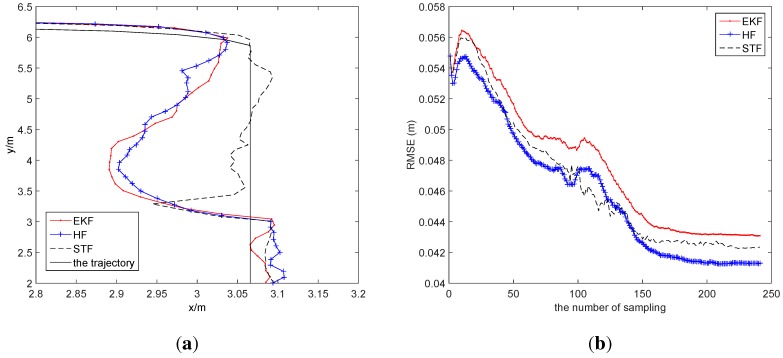
The impact of noise. (**a**) The tracking with noise; (**b**) the RMSE.

For investigating the robustness of the three schemes, we artificially add the disturbance, which is a π/4 orientation error, at the 90th sampling time step. Then, the average localization error of EKF is [0.0367 m, 0.0700 m, 0.0525 (rad)], the one of HF is [0.0380 m, 0.0739 m, 0.0570 (rad)] and the one of STF is [0.0300 m, 0.0702 m, 0.0369 (rad)]. In Figure 11a, STF clearly has a better tracking performance than EKF and HF. In Figure 11b, the curves of the RMSEs of EKF and HF make a sharp change at the 90th time step, while the curve of STF makes a consecutive change at the same time step, which demonstrates STF to be the most robust algorithm of the three methods.

**Figure 12:**
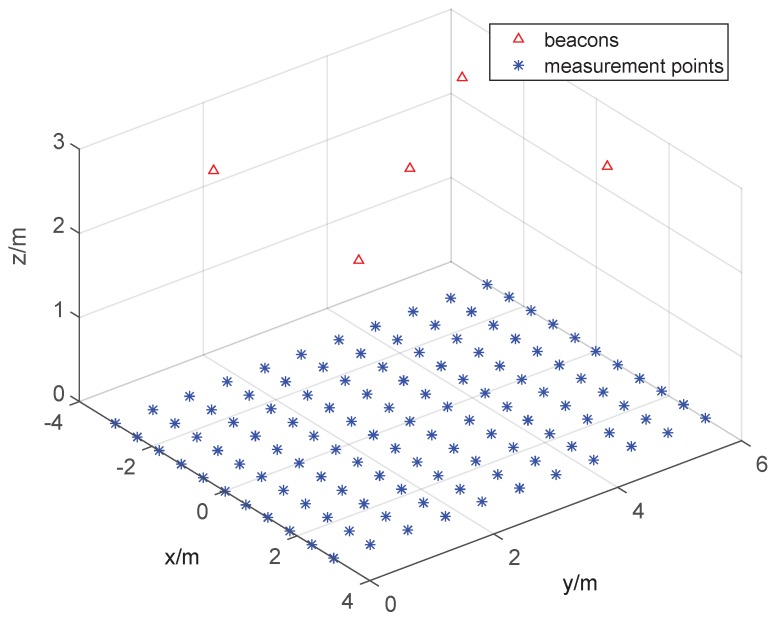
The configuration of the simulation.

Lastly, we assess the localization accuracy of the localization system by simulation, the configuration of which is the same as described in the previous section. The only difference is that there are 121 measurement points deployed in a 6 cm × 6 cm square region. The diagram is shown in Figure 12.

**Figure 13:**
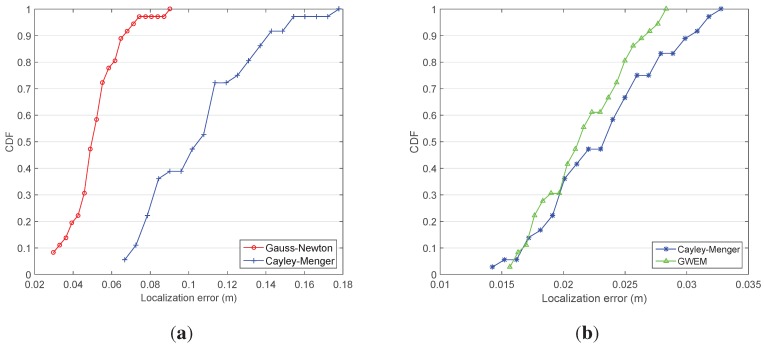
The comparison of CDF. (**a**) Gauss–Newton and Cayley–Menger; (**b**) Cayley–Menger and GWEM.

For evaluating the algorithm accuracy and calculation efficiency of the Gauss–Newton iterative method and the Cayley–Menger determinant, three randomly-picked beacons are involved in the simulation. The calculation duration of the Gauss–Newton iterative method is 420.1 s, equal to 0.58 s per iteration, and the calculation duration of the Cayley–Menger determinant is 0.68 s, equal to 0.9 ms per iteration. Undoubtedly, there is a vast difference of calculation efficiency between the two algorithms. In Figure 13a, we compare the CDF of the two algorithms, and it is illustrated that the Cayley–Menger determinant is of high localization efficiency, but low localization accuracy. The Gauss–Newton iterative method is the opposite.

If the five beacons are all involved in the calculation, the proposed GWEM is capable of fusing the redundancy information into the existing methods. In Figure 13b, GWEM has a better localization performance than the Cayley–Menger method. The average loop time of the former is 10.8 s, while the average loop time of the latter is 10.2 s, which demonstrates that GWEM has the tantamount efficiency compared to the Cayley–Menger method.

**Figure 14:**
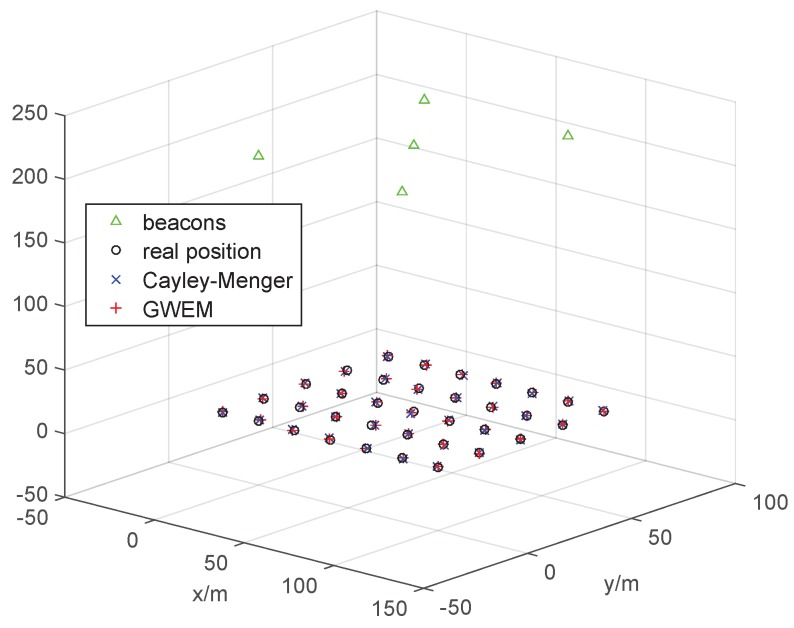
The experiment results.

**Figure 15:**
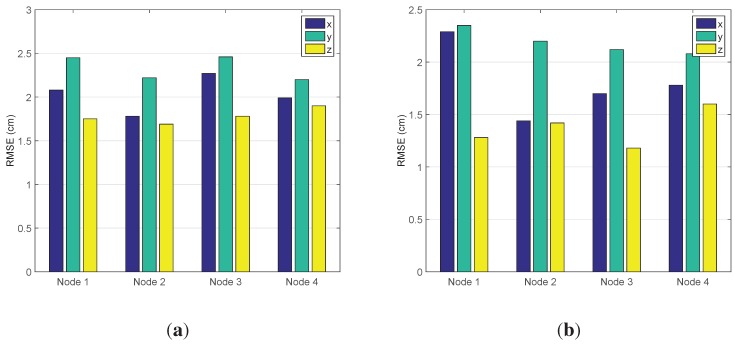
The comparison of the average error. (**a**) The average error of Cayley–Menger; (**b**) the average error of GWEM.

After simulation, we conduct the experiment to verify the proposed algorithms. In the experiment, the localization results are the average of the consequences of 20 trials by means of the Cayley–Menger method and GWEM, respectively, and the results are presented in Figure 14. For clarity, the histogram of the localization error of the two methods is shown in Figure 15. Comparing Figure 15a, and b, it is not difficult to find that GWEM has the lower average error. In the experiment, GWEM has been proven to be a fast and effective indoor localization method.

In our 3D localization system, when a robot carrying a node enters the 3D WSN, it can help unknown beacons on the ceiling determine their own positions; meanwhile, the locations of beacons can be calibrated further based on the robot’s trajectory. After the above process, the initialization of the localization system is done, and when the target with a node enters the system again, the system will calculate and decide the trajectory of the target precisely. Therefore, the self-localization and calibration of beacons are the pre-processing task, and the computational cost almost reduces to zero because all of the beacons’ locations are known [39]. When tracking targets, all of the measurements will be delivered to the PC terminal via the sink node; then, the data are processed in MATLAB. The computational complexity is approximately equal to a particle filter in the simulation.

## 8. Conclusions

The paper provides a series of feasible schemes for localization in 3D settings based on WSNs, which solves three essential problems: the self-localization of beacons, the calibration of beacons after self-localization and positioning and tracking the mobile target in 3D settings by beacons. Aimed at the three problems, the contributions of the paper are as follows. Firstly, the weighted least squares estimation of localization is proposed for self-localization of beacons, which minimizes the influence from measurement errors by means of DSA. Secondly, for higher accuracy, we employ the calibration scheme for beacons with the aid of the mobile robot. Then, after comparing the EKF, HF and STF methods, we conclude that HF has the best adaptiveness to the uncertain state model, and STF has the best tracking performance to the system with great disturbance. Thirdly, analyzing the attributes of the Gauss–Newton iterative method and the Cayley–Menger determinant, we propose the optimal node selection scheme based on GDOP, which can select the group of beacons with the minimum GDOP from all of the beacons. Then, GWEM is presented for fusing more information from other beacons. Lastly, the simulation and experiment are used for evaluating the proposed methods, and the consequences show that the methods are feasible for localization in 3D settings and have high localization accuracy.
